# Supplementary material for: The Longitudinal Association between Co-Residential Care Provision and Healthcare Use among the Portuguese Population Aged 50 and Over: A SHARE Study
Source: Int J Environ Res Public Health. 2023 Feb 23;20(5):3975. doi: 10.3390/ijerph20053975 (PMC10001838; doi:10.3390/ijerph20053975)
Supplement: Supplementary file 1 [file ijerph-20-03975-s001.zip › ijerph-2226676-supplementary.pdf]

Table S1- Statistic values of the Variance Inflation Factor (VIF) and Tolerance for Model 2.

Model 2	VIF	95% CI	Tolerance	95% CI
Co-residential care	1.06	[1.03-1.11]	0.95	[0.90-0.97]
Gender	1.12	[1.09-1.18]	0.89	[0.85-0.92]
Age	1.62	[1.55-1.70]	0.62	[0.59-0.65]
Education (ISCED)	1.27	[1.22-1.32]	0.79	[0.75-0.82]
Current job situation	1.50	[1.44-1.58]	0.67	[0.63-0.70]
Financial distress	1.16	[1.12-1.21]	0.86	[0.83-0.90]
Chronic diseases	1.19	[1.15-1.24]	0.84	[0.80-0.87]
Depressive symptoms	1.18	[1.14-1.24]	0.85	[0.81-0.88]
Physical inactivity	1.13	[1.09-1.18]	0.89	[0.85-0.92]
Hospitalized in the last 12 months	1.03	[1.01-1.10]	0.97	[0.91-0.99]
Household size	1.06	[1.03-1.12]	0.94	[0.90-0.97]
Social activities	1.21	[1.17-1.27]	0.82	[0.79-0.86]
Time	1.09	[1.06-1.14]	0.92	[0.88-0.95]

Table S2 - Longitudinal association between co-residential care provision and healthcare use among the Portuguese population aged 50 and over (Model 4: interaction term between *providing co-residential care* and *gender)*

	**Model 4**	
	**IRR (95% CI)**	***P - Value***
(Intercept)	1.69 (1.21 - 2.36)	**0.002**
**Co-residential care**		
Non-co-residential caregivers	ref.	
Co-residential spousal caregivers	0.95 (0.78-1.15)	0.591
Non-spousal co-residential caregivers	1.02 (0.85-1.21)	0.860
**Age**	1.00 (1.00-1.01)	0.127
**Sex** (Male)	0.93 (0.85 - 1.01)	0.076
**Current job situation (Retired)**	1.05 (0.96 - 1.14)	0.325
**Education (ISCED)**		
Low education level	ref.	
Medium education level	1.02 (0.89 - 1.17)	0.765
High education level	1.24 (1.09 - 1.42)	**0.001**
**Financial distress** (Yes)	1.07 (0.99 - 1.15)	0.107
**Hospitalized in the last 12 months** (Yes)	1.71 (1.56 - 1.89)	**<0.001**
**Chronic diseases**	1.14 (1.12 - 1.16)	**<0.001**
**Depressive symptoms (4 or more)** (Yes)	1.26 (1.17 - 1.35)	**<0.001**
**Physical inactivity** (Yes)	1.15 (1.06 - 1.25)	**0.001**
**Household size**	0.97 (0.94 - 1.00)	**0.054**
**Social activities**	0.98 (0.96 - 1.01)	0.254
**Time (wave)**	1.07 (1.04 - 1.10)	**<0.001**
**Spouse co-residential caregiver * Gender**	1.07 (0.82 - 1.40)	0.612
**Co-residential caregiver of other than a spouse * Gender**	0.94 (0.69 - 1.28)	0.689
Random Effects		
σ (intercept)	***0.3126***	
Goodness-of-fit		
**AIC**	16250	
**Deviance**	16210	
**Log-Likelihood**	-8105	
ICC	0.406	
Nº of observations	***3142***	
Nº of individuals	***1928***	
